# COPD management as a model for all chronic respiratory conditions: report of the 4^th^ Consensus Conference in Respiratory Medicine

**DOI:** 10.1186/s40248-017-0109-0

**Published:** 2017-11-10

**Authors:** Stefano Nardini, Fernando De Benedetto, Claudio M. Sanguinetti, Salvatore Bellofiore, Stefano Carlone, Salvatore Privitera, Luciano Sagliocca, Emmanuele Tupputi, with the collaboration of The Consensus Conference Group, Claudio Baccarani, Gennaro Caiffa, Maria Consiglia Calabrese, Antonio Capuozzo, Salvatore Cauchi, Valentina Conio, Giuseppe Coratella, Franco Crismancich, Roberto W. Dal Negro, Franco Dellarole, Maurizio Delucchi, Carlo Favaretti, Silvia Forte, Franca Matilde Gallo, Riccardo Giuliano, Marco Grandi, Antonino Grillo, Maria Rosaria Gualano, Enrico Guffanti, Salvatore Locicero, Francesco Paolo Lombardo, Marco Mantero, Roberto Marasso, Laura Martino, Michele Mastroberardino, Carlo Mereu, Roberto Messina, Margherita Neri, Bruno Franco Novelletto, Paolo Parente, Sergio Pasquinucci, Massimo Pistolesi, Mario Polverino, Agnese Posca, Luca Richeldi, Fernando Roccia, Ettore Saffi Giustini, Michelangelo Salemi, Salvatore Santacroce, Mario Schisano, Matteo Schisano, Eleonora Selvi, Andrea Silenzi, Patrizio Soverina, Claudio Taranto, Marta Ugolini, Piero Visaggi, Alessandro Zanasi

**Affiliations:** 1 Italian Respiratory Society - Research Center, Milan, Italy; 2 Italian Respiratory Society - Executive Committee, Milan, Italy; 3 AOU “Policlinico - Vittorio Emanuele”, Catania, Italy; 4 Italian Respiratory Society, Milan, Italy; 5 General Secretary Italian Respiratory Society, Milan, Italy; 6 Local Health Unit Salerno, Salerno, Italy; 7 General Secretary/Treasurer / Italian Respiratory Society-Research Center, Milan, Italy

**Keywords:** Chronic obstructive pulmonary disease, Organization, Responsibilities, Sustainability, Training

## Abstract

**Background:**

Non-communicable diseases (NCDs) kill 40 million people each year. The management of chronic respiratory NCDs such as chronic obstructive pulmonary disease (COPD) is particularly critical in Italy, where they are widespread and represent a heavy burden on healthcare resources. It is thus important to redefine the role and responsibility of respiratory specialists and their scientific societies, together with that of the whole healthcare system, in order to create a sustainable management of COPD, which could become a model for other chronic respiratory conditions.

**Methods:**

These issues were divided into four main topics (Training, Organization, Responsibilities, and Sustainability) and discussed at a Consensus Conference promoted by the Research Center of the Italian Respiratory Society held in Rome, Italy, 3–4 November 2016.

**Results and conclusions:**

Regarding training, important inadequacies emerged regarding specialist training - both the duration of practical training courses and teaching about chronic diseases like COPD. A better integration between university and teaching hospitals would improve the quality of specialization. A better organizational integration between hospital and specialists/general practitioners (GPs) in the local community is essential to improve the diagnostic and therapeutic pathways for chronic respiratory patients. Improving the care pathways is the joint responsibility of respiratory specialists, GPs, patients and their caregivers, and the healthcare system. The sustainability of the entire system depends on a better organization of the diagnostic-therapeutic pathways, in which also other stakeholders such as pharmacists and pharmaceutical companies can play an important role.

## Background

According to WHO [1], non-communicable diseases (NCDs) “kill 40 million people each year”. Globally, this figure represents about 70% of all deaths. Among NCDs, after cardiovascular diseases and cancer, respiratory diseases rank as the 3^rd^ cause of death, just before diabetes. Overall, more than 80% of all premature NCD deaths are due to these four diseases. In its action plan [2] WHO recommends “to strengthen and orient health systems to address the prevention and control of NCDs” and fosters a comprehensive approach to cope with them, which includes “detecting, screening and treating these diseases, and providing access to palliative care for people in need”.

The management of chronic diseases is particularly critical in the respiratory field. In Italy, these diseases are very widespread (respiratory diseases including lung cancer constitute the 2^nd^ cause of mortality) and account for high healthcare resources consumption. Nevertheless, throughout Italy the number of hospital respiratory units and specialist beds continues to decline (the number had already been reduced) without a consistent and organized alternative provided outside the hospital, while each year the number of respiratory specialists that graduate from universities is inadequate to meet the needs of the respiratory specialty.

In response to this situation, a redefinition of the contents of Respiratory Medicine is necessary, as well as of the role and responsibility of respiratory specialists and their scientific societies, recognizing the needs, and jointly sharing the organization of a new model of management of respiratory diseases for clinical practice that is sustainable by the whole system. The challenge is to render the care process effective and sustainable through the cooperation of all stakeholders, by: i) improving the prescriptive appropriateness and efficacy of general practitioners (GPs); ii) defining integrated care pathways involving GPs, respiratory specialists and the hospital; iii) developing new modalities of drug delivery; iv) promoting greater participation of patients and their care givers (in Italy, the model to follow is that of diabetes [3]) also with the use of telemedicine [4]; and v) involving the pharmaceutical system in the management of the whole process.

Chronic obstructive pulmonary disease (COPD) may be used as a model to discuss these issues. The prevalence of COPD is not known with certainty in Italy, but it is estimated to vary from 4.03 to 5.55% in males and 2.60 to 4.45% in females [5]. However, the results of a more recent survey in the general population, limited to North-Eastern Italy and based on a questionnaire and spirometry, revealed a global COPD prevalence much higher, ranging from 6.8 to 11.7% according to the screening method [6]. In any case, the disease is very widespread and has a heavy impact on the national health system (NHS). Indeed, several care models already exist, some of which have been adopted at local level, at least in a pilot phase.

To seek and propose solutions to the current challenge of chronic respiratory diseases in Italy, in particular COPD, the Research Center of the Italian Respiratory Society (IRS) organized the 4^th^ Consensus Conference in Respiratory Medicine, held in Rome, November 3–4, 2016, inviting the audience (all invited stakeholders) to discuss the issues and arrive at agreement on how to create a sustainable management of COPD, which could become a model for other chronic respiratory conditions. This paper is a report of what was discussed and agreed during the Consensus Conference.

## Methods

The Research Center of the Italian Respiratory Society (IRS) adopted the method of Consensus Conference, now in its Fourth Edition, in order to seek and propose solutions to the four topics:

1. *Training*: basic educational curriculum and continuing medication education (CME); the role of Institutions and Scientific Societies.
2. *Organization*: specific roles of the hospital, GPs, local community, and pharmaceutical system in a network of respiratory medicine; to define the role of policy makers, health professionals, and users in building up a network.
3. *Responsibilities*: the reciprocal responsibility of respiratory specialists, GPs, policy makers, patients and their caregivers.
4. *Sustainability*: epidemiological burden of respiratory diseases: primary prevention, early diagnosis, therapy, and rehabilitation. Sustainability of respiratory specialty in the NHS; definition of essential care levels and diagnostic-therapeutic pathways.

The Consensus Conference (CC) was organized and carried out according to a methodology recommended by the National Guidelines System (http://www.snlg-iss.it/cms/files/manuale_metodologico_consensus.pdf-). A CC is considered the most adequate tool for formulating recommendations on controversial topics that cannot be evaluated based on reviews and quantitative studies due to the many experiences that need to be confronted. However, usually the CC deals with technical or clinical aspects and the audience involved is composed of specialists of the same specialty. This CC dealing with organizational as well as political aspects required participation of representatives from different disciplines and backgrounds. Although some difficulties were experienced, after lively discussion a number of issues were formulated and then translated into recommendations. These were then reviewed and validated by the members of an independent Jury. Due to the nature of the topics treated, the expected result may be defined as the best possible agreement among the individual positions and experiences of participants, the group of experts and the Jury.

In detail, the players involved and their specific tasks were:

- The Promoting Committee (PC): included members of the Research Center of the Italian Respiratory Society, A.GE.NAS (National Agency for Health Assistance), Federanziani (Federation of Elderly), FIMMG (Italian Federation of General Practitioners), Health Ministry, SIMM (Italian Society of Medical Managers), and Federfarma (Federation of Pharmaceutical Industries). The PC promoted the CC, appointed the members of the Technical and Scientific Committee and those of the Jury, and prepared specific questions on each of the four selected topics.
- The Technical and Scientific Committee (TSC): composed of individuals of recognized experience in the field, discussed the questions and selected the teams charged with preparing the material for discussion during the conference.
- The Jury: composed of 12 members selected by the PC and TSC representing physicians (multidisciplinary context), patients and their associations. The Jury read the reports, attended the CC and participated in the discussion, recorded relevant new contributions to the presentations (including disagreement), and wrote up the consensus document.
- The Assembly: included all the people invited and who attended the CC.

## Results and discussion

The results for the four topics are reported below. Each topic was introduced by a brief review of the state of the art, according to recommendations of guidelines and organizational documents, followed by a comparison with the real situation existing now. The possible deviations and critical issues were outlined and some proposals were formulated for improving the management of COPD and other chronic respiratory diseases. At the end of the discussion on each topic, the assembly approved some statements, for subsequent examination by the Jury before definitive validation.

### Training

The questions preliminarily put to the Working Group in charge of developing this topic were:

- **Does the University adequately educate the specialist? If not, what are the main gaps in formation?**

Based on the report and lively discussion, the points that emerged and were approved by the Assembly were:

- University education for undergraduate medical students in Italy is substantially adequate concerning the rudiments of respiratory medicine.
- Fundamental notions of respiratory medicine are, however, not always taught by respiratory specialists but by others (e.g. by internists) with a resulting lower characterization and in-depth analysis of specialist notions, especially those relative to respiratory physiology, that are indispensable for a basic education.
- There are important inadequacies in the organization of students’ practical training, both in terms of the duration (which is too short and dispersed - i.e. few hours, differently scheduled in different universities – compared to the ideal duration of 1 month of theoretical and practical teaching linked to cardiology and thoracic surgery), and the training given on the most frequent chronic diseases (asthma and above all COPD, and cardiovascular comorbidities which frequently occur in these patients). Also, a strong need was stressed for organized teaching networks which include teaching hospitals (see below).

b. **Do non-academic hospitals have a role in the education of the specialist? If yes, what role and the timing?**

- The proposal made by academic respiratory specialists was confirmed on the need to set up teaching networks in which training is integrated with the so-called “teaching hospitals”, i.e. hospitals characterized by a high level of specialization, with expertise in other specialist or ultra-specialist areas associated to mainline respiratory medicine.
- This integration should be through practical training courses (e.g. mannequin maneuvers, tutor-assisted trials of mechanical ventilation, intubation, endoscopy etc.) that are certified and validated, carried out over the entire duration of specialization and with an adequate number organized each year.
- A similar training integration should be set up for chronic respiratory diseases like asthma and COPD (which are so widespread and have high rates of morbidity, mortality, and social costs), covering not only the acute (and semi-intensive) phase, but also the clinical and rehabilitative management outside hospital.
- At present, one of the most important issues in COPD management concerns the role of GPs, whose task is early diagnosis by means of anamnestic and clinical evaluation and spirometry. In fact, basic spirometry (first level investigation) could be carried out by GPs in their setting after adequate training, but this issue is still a matter of discussion and disagreement, while the specialist’s role would be a more in-depth patient assessment with second level examinations.
- Another critical point is related to the community health organization, still highly deficient as regards patient management and rehabilitation, which is important for health-related quality of life and long-term outcome of chronic patients.

c. **Besides the present model of Continuing Medical Education (CME), would another system based on certification both of the specialist and structure, with internal and external audit, be desirable? If yes, what and how often?**
  The HERMES Project (Harmonized Education in Respiratory Medicine for European Specialists) [7] could be adopted in Italy to educate respiratory specialists so that they are able to practice also in Europe or in other countries. The Assembly unanimously expressed its approval of this proposal, also hoping for an even greater inclusion of Italian specialists in high-level educational projects like HERMES under the aegis of the European Respiratory Society. However, this is not the only model and scientific societies could play an important role in delivering CME and carrying out audits. There is need for periodic evaluation of structures, specialists, and even GPs. HERMES is a European reference model already existing, but besides this the assembly pointed out the need for frequent internal and external audit at different levels in order to make our future organization more efficient and adequate.

### Organization

Questions relative to the second topic discussed were:

- **a) Not all Italian regions include in their health system the community respiratory specialist; is there any role for this type of specialist?**

After a lively discussion on this issue, the following statements were formulated:

1. The role of the outpatient specialist should be to cooperate with GPs in primary care settings for the diagnosis of diseases, promotion of primary and secondary prevention, and care of patients discharged from hospital to integrated home-care, and to be in charge of the respiratory rehabilitation program and end of life care.
2. To the hospital and community respiratory specialists are reserved: second and third level investigations for proper diagnosis and staging, clinical-therapeutic management of the acute phase, including in-hospital sub-intensive care, and the respiratory rehabilitation carried out inside or outside hospitals.
3. In Italy, the community respiratory specialist is not defined as a standard and there are still many health districts without such a physician. This kind of specialist can act as an intermediate reference figure between the hospital specialists and GPs. This figure would be of particular importance to take care, together with the GP, of those cases that can be treated in the community, so lowering the present heavy burden of hospital admissions.
4. It is believed that the presence of this type of specialist, included in a well-organized network of primary care, where the objectives are agreed and shared by all involved, could lead to a partial but significant decrease in hospital admissions.

- **b. The Italian (autonomous respiratory unit) and the United Kingdom (a multidisciplinary department with one senior and one junior consultant plus physicians in training) models of hospital specialist system: what are the points of strength and weakness?**

The conclusions of the discussion on this point were:

Apparently, there is no difference because both systems seem to meet the new health needs. Both in UK and in Italy (in both countries the public health system is going through an economic crisis) the organization of hospital structures does not seem coherent with the changes that have determined various new needs and demands on public health. The longer life expectancy and the change toward healthier lifestyles have led to an increased prevalence of chronic conditions, whose diagnostic and therapeutic management is totally different from that of acute illnesses. Presently chronic diseases represent 80% of all health requests [8], thus it is crucial to foster an improved quality of care in respiratory hospital departments, whether complex or simple units, for acute patients and manage the care of chronic diseases in the home setting. For this reason, the number of beds is not important but rather how they are utilized (i.e. only for acute patients requiring highly specialized care and for the shortest time possible, so creating a more efficient turnover). The assembly pointed out the need for some beds of intensive and/or semi-intensive care in each respiratory department (2–4 beds each 200,000 inhabitants). It is also important from a therapeutic point of view to organize community-based continuity of care and make respiratory post-intensive rehabilitation available in structures in the local community or at home. The assembly concluded that the Italian system better meets the health needs of the population than the UK system and seems more suitable as a model of specialized hospital respiratory care in future years, but it also recognized that the national health system, mainly to reduce costs, is massively reducing the number of respiratory units in Italy.

- **b. Some Italian regions have produced diagnostic-therapeutic pathways (DTPs) for COPD: what are the organizational similarities and differences?**

After an intense exchange of views and experiences by participants, an agreement was reached on these issues:

- Regarding regional and local diagnostic-therapeutic pathways, great differences emerged, especially concerning COPD, where the present situation varies from regions where all or nearly all local health systems have a specific DTP to other regions where DTPs are totally missing, or alternatively different denominations are used to identify the same organizational pathway. In some cases, the National Guidelines [9, 10] are not even mentioned.
- Not all give the necessary importance to primary prevention (lifestyle modification, etc.), smoking cessation treatment, or early diagnosis.
- The patient’s role is almost never indicated, while exclusive importance is assigned to the role of physicians and other health workers who take care of patients.
- The organization in the local community is different not only because the structure is different but mainly because at the moment much is based on the personal initiative of specialists, as a precise structural organization for national and local management is still lacking.
- The same competence is assigned to different figures (e.g. in some settings, performance of the first spirometry examination is the responsibility of the respiratory specialist, in others of the GP).
- In many settings some professional figures (e.g. nurse and physiotherapist) are missing that provide an appropriate and fundamental support for the specialist during the diagnostic-therapeutic-rehabilitative process of respiratory patients.
- Frequently, a rehabilitative pathway is not present or envisaged.

From these evaluations, it stems that not always is the community able to take care of patients who are in a post-acute phase, and to manage the whole pathway of chronic respiratory disease at home. This seems to be the main reason why hospital admissions for COPD are so numerous.

Rehabilitative structures, post-acute departments, associations of GPs, domiciliary nurse assistance, active patient associations, and a suitable family environment are all lacking, and, most important, there is no definition of network fundamentals (agreed and shared roles and interventions of the health personnel who are in touch with the patients). The results reported at point “a” and “b” of this session are an attempt to respond to these inadequacies and could be implemented according to the whole COPD pathway described in a recent multidisciplinary paper [11].

### Responsibilities

In this session the questions to be discussed were:

- **What is the responsibility of the respiratory specialist for the management of chronic respiratory disease?**
- **What is the responsibility of the GP for the management of chronic respiratory disease?**
- **What is the responsibility of patients and their caregivers for the management of chronic respiratory disease?**

The Assembly agreed that the adherence to therapy and the integration of care are indispensable to reduce the organizational deficiencies underlying inappropriate care practices. These can be measured everywhere in our country by the rate of unsuitable admissions to emergency departments (white codes) of patients affected with chronic diseases. Obviously, the emergency department, although providing an immediate practical solution, does not resolve the problem of continuity of care, but rather renders it more complicated. In fact, while it copes with a health need (true or supposed) at that moment, it further fragments the care pathway. Conversely, to obtain the best management of COPD it is necessary to make GPs more responsible. The solution could be to create intermediate levels of assistance between primary care and hospital, set up home and community care structures, and formulate a new definition of the hospital’s task as acute care only. The Assembly recognized that it is hard, if not impossible, to put this into practice if the NHS does not become strongly aware of these issues. In fact, it must plan and fund the activity of prevention and the integration of health services. Specifically, the NHS’s task is:

1. To organize the “respiratory care network” i.e. the coordination system between hospital and local territory, promote the setting up of DTPs (possibly similar to a National model which - at least for COPD - already exists [10]) suited to the reality and sustainable, and assess their feasibility and efficiency by means of audit and periodic reviews.
2. To organize and fund effective communication campaigns in order to inform people about chronic respiratory diseases, their characteristics and modes of prevention, early diagnosis and treatment adherence.
3. To promote and fund the computerization (according to a unique National model and consequently utilizable by any regional systems) of the health system making it possible to share in real-time, between interested (and authorized) subjects, all the health information related to an individual. Unfortunately, the present computerized information system is not uniformly established throughout the country, and there are frequent differences both among regions and among health districts of the same region. As well, the programs are not always able to dialogue each other or not suitable for a health environment and related issues.
4. To formulate a new definition of the timing of specialist consultations and services which would take into account the real time consumption of these services including not only the usual medical activities (history, diagnosis and prescriptions) but also education about the disease (nature and evolution of the disease), counseling and training in device use. This action may parallel that at point 3.
5. (partly alternative to the preceding point) To review tasks and competences, e.g. some activities presently carried out by respiratory specialists (e.g. filling in a form, education about the disease) should instead be carried out by administrative, health, or voluntary personnel, adequately trained. Hence it is necessary to promote a coherent education of all health workers to reach the above specific objectives.

In this context, the health professionals (both respiratory specialists and GPs) have the responsibility for:

1. Adherence (as per their specific competence) to the agreed diagnostic-therapeutic pathway for COPD and other chronic respiratory diseases. These should always be proposed, discussed and finalized together with all interested parties (including patients and their caregivers). To supervise that the official DTPs are the standard to which all make reference. For the adherence of GPs to the DTP, a ‘proactive medicine’ approach should be incentivized.
2. A novel task of collecting clinical data, besides the simple administrative ones, that would contribute to represent the “in the field” real-world activity. This data collection should also include audit initiatives.

Finally, patients, their caregivers, and associations, have the responsibility for:

1. An optimum adherence to therapy and in general to doctors’ prescriptions, after receiving the necessary education about disease and learning how to verify the progress. This duty to adherence includes corrective actions in the case of manifest failure to comply with the prescriptions or deviation from them. For example, one should be rigorous about suspending oxygen therapy if the patient, after being offered smoking cessation treatment, continues smoking.
2. A conscious and responsible use of health service resources, consisting in hospital admission only when strictly appropriate, with an increase in scheduled control visits and a decrease in unscheduled ones. In this respect, patient and caregivers must be aware of the diagnostic-therapeutic pathway, and should “called to account” when their behavior diverges from the predicted pattern, even if they are in non-emergency conditions.

### Sustainability

The final session of the Consensus Conference dealt with sustainability based on these questions:

- **Does the multidisciplinary approach really give support to sustainability?**

The answer would be ‘yes’ if care pathways can be designed that are supported by a network structure consisting of the following nodes (depending on the disease): hospital, community resources (GPs included), and social services. In this context, the continuity and appropriateness of care are the pillars on which to organize the pathways. This requires that the programs be patient-centered and include shared protocols based on common organizational principles set out in documents to consult according to the severity stage of the disease. The multidisciplinary approach could really be a substantial support to sustainability if the technical and professional path of the different care processes is correctly defined, i.e. the protocols to which the different professionals, each according to their own competence, must necessarily refer to.

b. **Does the pharmacist have a role in the sustainability of the system?**

The Assembly agreed that in a new organization aimed at sustainability, the pharmacist can play a fundamental role mainly as regards early diagnosis of COPD based on filling in questionnaires, but also by counseling patients to give up smoking and to maintain a correct life style, training them to use inhalation devices now available and to check periodically the appropriateness of their use, and controlling the adherence to therapy.

It was also recognized as a priority to develop and implement a valid program of specific respiratory education for pharmacists, including an accreditation process, as already performed in other countries like UK, Australia, and Canada [12, 13], which allows them to offer a high quality service that can meet the real needs of patients. Such an educational resource could contribute to improve the respiratory health of the Italian population through a greater adherence to therapy that could lead to a significant reduction in direct and indirect NHS costs. Therefore, the CC proposed that accredited pharmacies display a specific identification mark attached to the entrance of the pharmacy.

c. **What is the effective role of pharmaceutical industries and of pharmacotherapy for the sustainability of the system?**

Pharmaceutical industries could help in coping with COPD and other chronic respiratory diseases by improving quality of life and life expectancy through the development of new drugs that are more effective, more user-friendly and more sustainable than existing ones. It was pointed out that, besides the role that State and Health Service can play in terms of advocacy on chronic respiratory diseases, pharmaceutical industries can too play a fundamental role in education of people to health and of patients about their disease. To this end, internet sites and network resources can be used, clearly stating any conflicts of interest. There is little doubt that educating patients on different issues (e.g. improving their knowledge of risk factors, making them aware of the importance and efficacy of prevention, promoting educational campaigns, and stimulating and improving education about therapy) will allow a better control of the disease course and significantly reduce GP and respiratory specialist visits, admissions to the emergency department, ordinary hospital admissions, and the rate of lost working days or of inactivity.

## Conclusions

To help the NHS to cope with chronic respiratory diseases, the Research Center of the Italian Respiratory Society organized in 2016 the 4^th^ Consensus Conference on Respiratory Medicine. COPD was chosen as the paradigm of chronic respiratory disease care. Specialists in different specialties (clinical and managerial) discussed the main obstacles to an effective, safe and sustainable respiratory care and proposed the solutions to overcome them.

As for the education and training of health staff, the university education in Italy of medical students on respiratory medicine is currently sufficient. However, respiratory pathophysiology, social aspects of chronic conditions (like asthma and COPD) and practical training need to be improved. This can be achieved by forming an educational network linking the University with excellent hospital units (for interventional and emergency care) and forefront primary care structures (for chronic conditions assisted via community and population medicine).

To build a network aimed at providing integrated care for respiratory patients, the role of an outpatient respiratory specialist, i.e. a chest physician working (full or part time) outside the hospital, is capital. Such a physician could create an intermediate level of care to help GPs and the whole community in promoting primary prevention and early diagnosis (particularly of COPD) as well as managing chronic care post-hospitalization, rehabilitation and finally in providing end-of-life care. Although a network as described could help the NHS to minimize hospitalizations, in Italy no standards of education and no formal definitions of such a figure exist (neither do they exist, actually, for specialized nurses and physiotherapists able to work with the outpatient respiratory specialist).

In shaping the new model of respiratory care a new definition of responsibility of each stakeholder is necessary. GPs (educated by chest physicians during medical school about the fundamentals of respiratory medicine) need to know the first level diagnostic tools and how to manage them in prevention and treatment. Putting together a group of GPs in which at least one of them is specially interested in respiratory medicine could help.

Respiratory specialists have the responsibility to be educated and trained in second-level interventions. Their practice should include not only theoretical and practical issues about second-level respiratory medicine but also the ability to carry out audits on both own consistency and system effectiveness. The European Respiratory Society syllabus, as well as the ERS Hermes program, could lead the way here.

A responsibility shared by GPs and respiratory specialists is to collaborate to build up diagnostic and therapeutic pathways of care as standards of care. Such pathways and indeed all the actions needed for building the new model of care require a multidisciplinary approach which is a responsibility of all stakeholders. A model exists for COPD in Italy, built up by the Associations of Nationwide Health districts and Municipalities- Federsanità-ANCI [10].

The NHS should have the responsibility to organize the network, to pay for it and for the auditing of national and regional DTP, to subsidize not only proactive medicine but also data collection about different experiences. NHS should also provide resources to the community for health education on living in good health and to the patients and their caregivers tools for managing disease in the best way.

While citizens have the responsibility to know and practice the behaviors to prevent respiratory (and indeed all) chronic conditions as well as the characteristics of the early phases of respiratory diseases (to get confirmation or disconfirmation of them through the appropriate actions of secondary prevention), patients and caregivers should be educated to self-management of their disease with special attention to the proper use of inhalation devices.

This last point emphasizes the role of pharmacists - properly educated and trained - in the network. Pharmacists can help in early diagnosis, in drug intake monitoring (with special regard to drugs for comorbidities) and in monitoring adherence to treatment of respiratory conditions as well as adverse effects. Finally, pharmaceutical industries could help in coping with the epidemic of chronic respiratory diseases by making available new drugs, with the aims of improving quality of life and life expectancy. However, in our opinion, new drugs must not only satisfy needs still not satisfied but also do so in a sustainable way. In other words, new drugs should be more effective, more user friendly, and more sustainable than the existing ones [14]. In addition, Pharma industries can collaborate in the management of COPD and other chronic respiratory conditions by helping in advocating on chronic respiratory diseases and in educating the community to health and patients about their disease.
